# Efficacy of Online Goal Management Training for Age-Associated Executive Impairment

**DOI:** 10.1093/geroni/igab046.1670

**Published:** 2021-12-17

**Authors:** Lynn Zhu, Danielle D'Amico, Susan Vandermorris, Yushu Wang, Laryssa Levesque, Jordan Lass, Angela Troyer, Brian Levine

**Affiliations:** 1 Baycrest, Toronto, Ontario, Canada; 2 Ryerson University, Toronto, Ontario, Canada

## Abstract

Goal Management Training® (GMT) is a standardized cognitive rehabilitation program that enhances individuals’ awareness of executive function impairments and trains them to regularly monitor and manage their goals. In-person GMT is well-validated among numerous subpopulations, including people experiencing age-related cognitive impairment or acquired brain injury, and people with psychiatric disorders. The goal of this study was to evaluate the efficacy and usability of online GMT relative to computerized “brain training” in a registered randomized controlled trial (protocol NCT03602768 at Trials.gov). Both interventions were administered in a self-paced format, with background therapist support provided for GMT. Primary outcomes were measured as self-reported executive impairment on standardized measures (the Dysexecutive Questionnaire and the Cognitive Failures Questionnaire) at pre-, immediate post-, and 6 weeks post-intervention. 62 older adults without psychiatric or neurological diagnoses completed the trial (online GMT: n = 37, age[mean] = 69 years; computerized brain training: n = 25, age[mean] = 64 years; both groups: 76% female). Improvements on the primary outcomes were observed post-intervention and were maintained at follow-up. GMT and computerized brain training groups could not be differentiated statistically, possibly due to restriction of range in the outcome measures at baseline. Additionally, the self-paced format prolonged the intervention beyond the recommended duration, which may have diluted efficacy. GMT was well-received, with participants reporting frequent use of the trained metacognitive strategies. Future studies will examine online GMT’s effectiveness in samples with documented executive impairment and with additional supports to promote engagement for this virtual program.

